# Brassinosteroids mediate susceptibility to brown planthopper by integrating with the salicylic acid and jasmonic acid pathways in rice

**DOI:** 10.1093/jxb/ery223

**Published:** 2018-06-08

**Authors:** Gen Pan, Yuqiang Liu, Linshan Ji, Xiao Zhang, Jun He, Jie Huang, Zeyu Qiu, Daoming Liu, Zhiguang Sun, Tingting Xu, Linglong Liu, Chunming Wang, Ling Jiang, Xianian Cheng, Jianmin Wan

**Affiliations:** 1 State Key Laboratory for Crop Genetics & Germplasm Enhancement, Jiangsu Provincial Center of Plant Gene Engineering, Nanjing Agricultural University, Weigang, Nanjing, China; 2 Institute of Crop Science, the National Key Facility for Crop Gene Resources and Genetic Improvement, Chinese Academy of Agricultural Sciences, Beijing, China

**Keywords:** Brassinosteroids (BRs), jasmonic acid (JA), *Nilaparvata lugens* (BPH), *Oryza sativa* (rice), salicylic acid (SA)

## Abstract

Improved knowledge of the interactions between plants and insects will facilitate better insect control in crops. Brassinosteroids (BRs) play a vital role in plant growth, developmental processes, and responses to pathogen infection, but the role of BRs in interactions between plants and insects remain largely unknown. In this study, we characterized a negative role of BRs in rice defense against brown planthopper (BPH, *Nilaparvata lugens*) and examined its underlying mechanisms. We found that BPH infestation suppressed the BR pathway while successively activating the salicylic acid (SA) and jasmonic acid (JA) pathways. In addition, BR-overproducing mutants and plants treated with 24-epibrassinolide (BL) showed increased susceptibility to BPH, whereas BR-deficient mutants were more resistant than the wild-type. BRs down-regulated the expression of genes related to the SA pathway and reduced SA content while genes related to the JA pathway were up-regulated and JA content increased after BPH infestation. Furthermore, BR-mediated suppression of the SA pathway was impaired both in JA-deficient and JA-insensitive mutants. Our results demonstrate that BRs promote the susceptibility of rice plants to BPH by modulating the SA and JA pathways.

## Teaser

Brassinosteroids play a negative role in rice defense against infestation by the brown planthopper through contrasting effects on the salicylic acid and jasmonic acid pathways.

## Introduction

Rice (*Oryza sativa* L.) is one of the most important staple foods, feeding over half of the world population. Insect pests are a major threat to rice production. The brown planthopper (BPH; Homoptera: Delphacidae) is a typical phloem-sucking herbivore and is one of the most serious and destructive insect pests in rice-growing areas (Normile, 2008). As well as causing direct damage, it also indirectly affects plants by transmitting viruses, including the rice ragged stunt virus and the grassy stunt virus (Liu *et al.*, 2015). The most economical and efficient strategy to control BPH is host resistance (Jena *et al.*, 2006). Although at least eight BPH-resistance genes have been cloned, knowledge of the underlying molecular mechanisms of plant–BPH interactions is still limited (Du *et al.*, 2009; Liu *et al.*, 2015; Tamura *et al.*, 2014; Wang *et al.*, 2015*b*; Ji *et al.*, 2016; Ren *et al.*, 2016; Zhao *et al.*, 2016; Guo *et al.*, 2018).

Previous studies have indicated that interactions between plants and phloem-sucking herbivores share similar mechanisms to plant–pathogen interactions. For example, the BPH resistance locus *Bph3* in rice is a cluster of three genes encoding plasma membrane-localized lectin receptor kinases (*LecRK*s) that are considered to be potential cell-surface receptors that prime pattern-triggered immunity (PTI) responses (Liu *et al.*, 2015). Furthermore, *Bph9*, *Bph14*, *Bph18*, and *Bph26* as well as the aphid-resistance gene *Mi1.2* have been categorized as genes that produce the coiled-coil nucleotide-binding site and leucine-rich repeat motif proteins (CC-NB-LRR) that are known to mediate resistance through direct or indirect recognition of pathogen effectors in rice (Rossi *et al.*, 1998; Du *et al.*, 2009; Tamura *et al.*, 2014; Ji *et al.*, 2016; Liu and Wang, 2016; Zhao *et al.*, 2016).

Salicylic acid (SA) is a small-molecule plant hormone that plays a vital role in plant innate immunity against pathogens, and several studies have shown that it is involved in resistance to phloem-feeding insects. For example, aphid infestation can induce SA accumulation and increase the activity of phenylalanine ammonia-lyase (PAL), a key enzyme in SA biosynthesis in wheat and barley (Mohase and van der Westhuizen, 2002; Chaman *et al.*, 2003). The SA pathway has been reported as participating in *Bph9*-, *Bph14*-, and *Bph29*-mediated BPH resistance in rice, with the SA content accumulating after BPH infestation (Du *et al.*, 2009; Zhou *et al.*, 2009; Ye *et al.*, 2012; Wang *et al.*, 2015*b*; Zhao *et al.*, 2016). These findings indicate that SA plays an important role in plant defense response to sap-sucking herbivores.

Jasmonic acid (JA) is synthesized from linolenic acid through the action of several enzymes in plant chloroplast membranes, and current evidence indicates that it induces resistance against necrotrophic pathogens and chewing herbivores (Wasternack and Hause, 2013). Antagonistic interactions between SA and JA are well documented (Aljbory and Chen, 2018). Several studies have demonstrated SA-mediated suppression of JA through the action of the *NONEXPRESSOR OF PATHOGENESIS-RELATED PROTEINS1* (*NPR1*), *MYB44*, and *WRKY70* genes in Arabidopsis (Spoel *et al.*, 2003; Li *et al.*, 2004; Shim *et al.*, 2013). Other studies have shown that JA can inhibit SA through the *JAZ* and *MYC2* genes in Arabidopsis (Chen *et al.*, 2009; Lorenzo *et al.*, 2004). Both the SA and JA pathways in rice have been shown to be induced by BPH feeding (Du *et al.*, 2009; Ye *et al.*, 2012; Zhao *et al.*, 2016). However, SA content and BPH resistance were significantly increased when expression of *13-lipoxygenase*, *OsHI-LOX*, a JA biosynthesis-related gene was silenced (Zhou *et al.*, 2009). Hence, the relationship between SA and JA in the immunity response to BPH seems ambiguous, or paradoxical in at least some instances, and needs to be further investigated.

Brassinosteroids (BRs) are a class of steroid phytohormones that regulate many aspects of plant growth and development (Tong and Chu, 2012; Zhu *et al.*, 2015; Feng *et al.*, 2016; Wu *et al.*, 2016). Many studies have shown that BRs function as negative regulators of innate immunity in plants (Albrecht *et al.*, 2012; Belkhadir *et al.*, 2012; De Vleesschauwer *et al.*, 2012; Nahar *et al.*, 2013; He *et al.*, 2017). Interactions between BRs and SA have been reported in the disease-resistance response in rice (De Vleesschauwer *et al.*, 2012), and it has been found that the susceptibility mediated by BRs is also suppressed by the JA pathway (Nahar *et al.*, 2013; He *et al.*, 2017). In contrast to the relative wealth of understanding of the functions of BRs in the interactions of plants and pathogens, little information is available about the role of BRs in defense against herbivores, especially with regard to phloem-sucking herbivores in monocots.

To better understand the mechanisms of rice–BPH interactions, we investigated the effects of BPH infestation on the BR pathway. We found that it was significantly inhibited by infestation and that two BR-overproducing mutants showed higher susceptibility to BPH than the wild-type. In contrast, BR-deficient plants displayed increased resistance. Further investigation showed that the role of BRs in promoting BPH susceptibility might be mediated by suppressing SA-mediated defense. In addition, BR-mediated suppression of SA depended on the JA pathway. Based on our results, we propose that BRs mediate susceptibility to BPH in rice by modulating the SA and JA pathways.

## Materials and methods

### Plant materials and growth conditions

The rice lines (*Oryza sativa* L.) used in this work included *m107*, *slg-D*, *NahG*, *lhdd10*, *coi1-18*, *og1*, and Zhonghua 11. *m107* and *NahG* were kindly provided by Professor Chencai Chu (Institute of Genetics and Developmental Biology, Chinese Academy of Sciences, Beijing) (Tang *et al.*, 2011; Tong *et al.*, 2012). Both *m107* and *slg-D* are dominant mutants that overproduce BRs (Wan *et al.*, 2009; Feng *et al.*, 2016). *NahG* is a SA-deficient transgenic line heterologously expressing the bacterial salicylate hydroxylase gene. *coi1-18* is a JA-insensitive mutant kindly provided by Professor Donglei Yang (Nanjing Agricultural University, Nanjing) (Yang *et al.*, 2012). The wild-type (WT) of *coi1-18*, *m107*, and *NahG* is the variety Nipponbare, whereas the variety Dongjin is the WT of *slg-D*. *og1* is a JA-deficient mutant with a mutation in the JA biosynthesis-related gene *OPDA-reductase7* (*OsOPR7*) and *lhdd10* is a BR-deficient mutant that has a *brd2* allele (Liu *et al.*, 2016; Li *et al.*, 2018). Both *og1* and *lhdd10* have 9311 as the WT. All plants were cultivated in an experimental field at Nanjing under natural long-day conditions.

### Maintenance of the brown planthopper

A colony of BPH, collected from rice fields in Nanjing, was maintained on the BPH-susceptible variety Taichung Native 1 (TN1) in a greenhouse (16/8 h light/dark, 26–28 °C, 60% relative humidity).

### Evaluation of rice mortality in response to BPH

A bulk seedling test was conducted to evaluate the response of plants to BPH as previously described by Wu *et al* (2014). Seeds were pre-germinated to ensure that the seedlings were at the same growth stage for infestation. Approximately 30 seedlings were grown in 10-cm diameter plastic pots. When the seedlings were at the second-leaf stage, they were thinned to 25 plants per pot and infested with 2nd- to 3rd-instar BPH nymphs at a density of 10 insects per seedling. When the mortality rate of the susceptible control plants had reached 90%, the mortalities of the other cultivars and lines were recorded. At least three replicates were used for each cultivar and line.

### BPH preference test

One seed each of the WT and mutant was sown in a 10-cm diameter pot. When they were at the third-leaf stage, the plants in each pot were infested with 15 BPH nymphs (2nd- to 3rd-instar) and placed within plastic cages. The number of BPHs on each variety was recorded at 6, 12, 24, and 48 h post-infestation. The experiments were repeated 10 times.

### BPH survival rate test

BPH survival rate tests were performed as previously described with minor modifications (Zhou *et al.*, 2009). Pots containing one plant at the fourth-leaf stage were individually covered with plastic cages (diameter 6 cm, height 9 cm) into which 15 newly hatched BPH nymphs were released. The number of surviving BPH nymphs on each plant was recorded at 2, 4, 6, and 8 d after the introduction of the nymphs. The experiment was repeated eight times.

### Hormone treatments

24-epibrassinolide (BL) and SA (Sigma, USA) were dissolved in ethanol and diluted into the concentrations required for use. An equivalent amount of ethanol was used for control treatments. In experiments evaluating the effects of BL and SA on seedling mortality in response to BPH infestation, seedlings sown in 10-cm diameter plastic pots were sprayed with 1 µM BL, 10 µM BL, or 100 µM SA at the second-leaf stage. Infestation commenced 12 h after application of BL and SA. To investigate the effects of BL and SA on BPH survival rate, plants at the three- to four-leaf stage were sprayed with 1 µM BL, 10 µM BL, or 100 µM SA 12 h before infestation with 15 BPH nymphs per plant. The plants were sampled 24 h later for RNA extraction and hormone measurement (SA and JA).

### RNA extraction and qRT-PCR analysis

Total RNA was extracted from leaf sheaths of plants infested with BPH for 0, 6, 12, and 24 h together with mock- and BL-treated plants. A RNAprep Pure Plant Kit (Tiangen, Beijing) was used for RNA extraction. First-strand cDNA was reverse-transcribed from 1 μg of total RNA using a PrimeScript 1st Strand cDNA Synthesis Kit (TaKaRa, Japan). Quantitative RT-PCR (qRT-PCR) was performed using a SYBR Premix Ex TaqTM kit (TaKaRa) on an ABI Prism 7500 Real-Time PCR System according to the manufacturer’s instructions. The *Actin* gene was used as the internal control. Primers used for qRT-PCR analysis are listed in Supplementary Table S1 at *JXB* online. The experiment was repeated with at least two biological replicates and three technical replicates.

### Hormone measurements

The SA extracted from 0.1 g (FW) of leaf sheath of seedlings at 24 h after BPH infestation was quantiﬁed using ADPWH_*lux*, a biosensor strain of an *Acinetobacter* species, as described previously (Huang *et al.*, 2006; Wang *et al.*, 2014).

The levels of JA were determined by the Zoonbio Biotechnology Co., Ltd (Nanjing, China). Approximately 0.2 g (FW) of leaf sheath from BL-treated or mock-treated plants at 24 h after infestation with BPH were ground in a pre-cooled mortar that contained 2 ml extraction buffer composed of isopropanol/hydrochloric acid (1000:1). The extract was shaken at 4 °C for 30 min, then 4 ml dichloromethane was added, and the sample was again shaken at 4 °C for 30 min and centrifuged at 13 000 rpm for 5 min at the same temperature. The lower, organic phase was then extracted, dried under N_2_, dissolved in 400 µl methanol (0.1% methane acid) and filtered with a 0.22-mm filter membrane. The purified product was then subjected to HPLC-tandem mass spectrometry (HPLC-MS/MS) analysis. HPLC analysis was performed using a ZORBAX SB-C18 (Agilent Technologies) column (2.1 mm × 50 mm, internal diameter 3.5 mm). The mobile phase-A solvents consisted of methanol/0.1% methanoic acid, and the mobile phase-B solvents consisted of ultrapure water/0.1% methanoic acid. The injection volume was 2 ml. MS conditions were as follows: the spray voltage was 4500 V; the pressure of the air curtain, nebulizer, and aux gas were 15, 65, and 70 psi, respectively; and the atomizing temperature was 400 °C.

Quantification of endogenous BRs (castasterone and 6-deoxocastasterone) was performed as described previously Ding *et al.* (2013). About 4 g (FW) of seedling stems from plants at 24 h after BPH infestation were harvested for BR measurements, together with stems from uninfested controls.

### Statistical analysis

One-way, two-way, and repeated-measures ANOVA tests, and binomial exact tests were performed using IBM SPSS Statistics version 20 software (SPSS Inc., Chicago, IL).

## Results

### BPH infestation inhibits the BR pathway and activates the SA and JA pathways

**Fig. 1.**
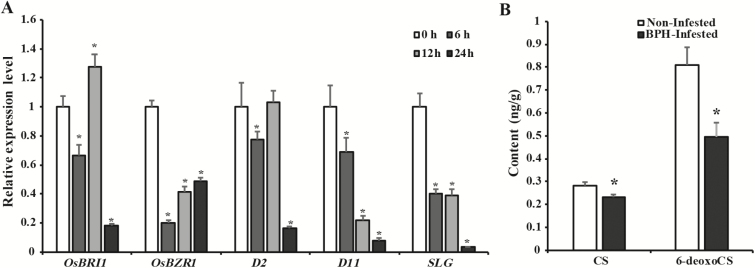
Effects of brown planthopper (BPH) infestation on the brassinosteroid (BR) pathway in rice. (A) Quantitative reverse-transcription PCR (qRT-PCR) analysis of BR-related genes (*OsBRI1*, *OsBZR1*, *D11*, *SLG*, and *D2*) in rice. Leaf sheaths of 2-week-old Zhonghua 11 plants at 0, 6, 12, and 24 h after infestation with BPH were used for analyses, with *Actin* as the internal reference gene. Data are means (±SD), *n*=3. The expression level at 0 h was set as 1.0. Significant differences compared with the expression level at 0 h were determined by one-way ANOVA with *post hoc* contrasts by Tukey test: *P<0.05. (B) Levels of castasterone (CS) and 6-deoxocastasterone (6-deoxoCS) in rice stems of non-infested and BPH-infested plants. Data are means (±SD), *n*=3. Significant differences were determined by one-way ANOVA: *P<0.05.

To determine the role of BRs in rice defense against BPH, transcript levels of BR pathway-related genes were determined following BPH infestation. qRT-PCR showed that expression levels of the BR receptor *BR INSENSITIVE 1* (*BRI1*) and the BR signaling component *BRASSINAZOLE RESISTANT 1* (*BZR1*) were decreased, especially at 24 h post-BPH infestation (Fig. 1A). Similar expression patterns were also detected for *SLG* and the BR-biosynthesis related genes *D11* and *D2* (Zhi *et al.*, 2003; Tanabe, 2005; Feng *et al.*, 2016). To confirm the effect of decreased expression of BR biosynthesis-related genes on the endogenous BR content, we analysed concentrations in BPH-infested plants at 24 h post-infestation. As reported in Chung and Choe (2013), rice plants do not show detectable levels of BL; castasterone (CS) is most likely an end product in rice, and 6-deoxocastasterone (6-deoxoCS) is a precursor for CS. The CS concentration in stems of BPH-infested plants was decreased compared with that in uninfested controls (0.23 ng g^–1^ versus 0.28 ng g^–1^ FW) (Fig. 1B). In addition, the 6-deoxoCS concentration in non-infested plants was 0.81 ng g^–1^ FW while in infested plants it was 0.49 ng g^–1^ FW (Fig. 1B). These results indicated that the BR-dependent pathway was inhibited by BPH feeding in rice.

As the SA and JA pathways have been widely shown to have roles in defense responses of plants to insects (Fürstenberg-Hägg *et al.*, 2013; Aljbory and Chen, 2018), genes related to these pathways were also investigated. As shown in Supplementary Fig. S1A, the SA biosynthetic genes *isochorismate synthase 1* (*OsICS1*) and *phenylalanine ammonia-lyases* (*OsPAL*) were markedly induced by BPH at both 6 and 12 h post-infestation, followed by a decrease at 24 h. Moreover, *OsNH1* (a homolog of *NONEXPRESSOR OF PATHOGENESIS-RELATED GENES 1* in Arabidopsis) and *OsPR5* (a SA-responsive gene) were also slightly induced. Two JA biosynthesis-related genes, lipoxygenase (*OsLOX1*) and allene oxide synthase (*OsAOS2*), were significantly increased at 24 h post-infestation. Similar up-regulated expression was also detected for the JA signaling-related genes *OsJAmyb* and *OsMYC2* (Supplementary Fig. S1B). Overall, these results indicated that the BR pathway was inhibited by BPH feeding. In contrast, the SA pathway was rapidly activated, followed by a decrease. The JA pathway was induced following activation of the SA pathway, which was similar to the results found in a previous study (Zhou *et al.*, 2009).

### BRs promote susceptibility to BPH infestation

**Fig. 2.**
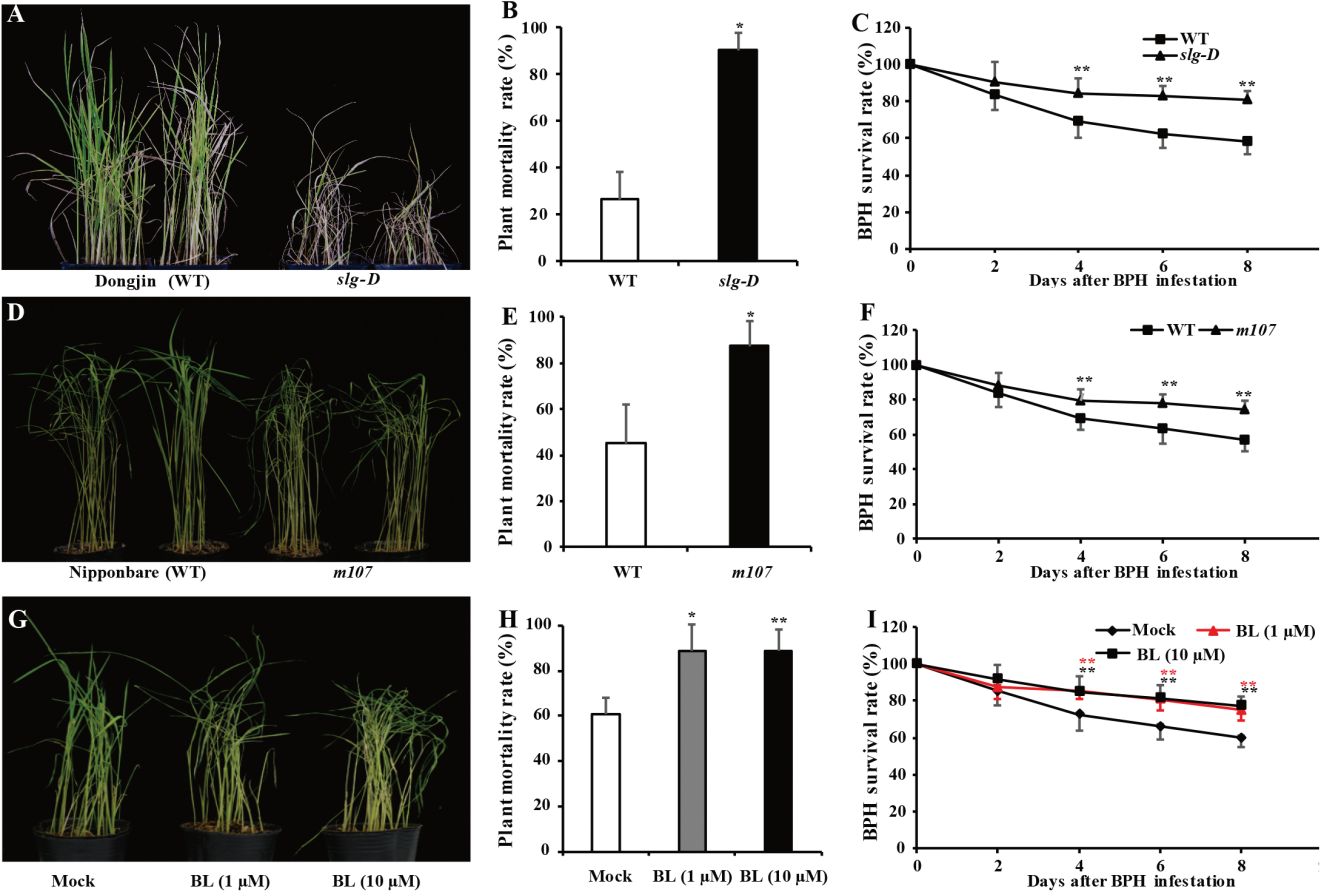
Brassinosteroids (BRs) promote susceptibility to brown planthopper (BPH) in rice. (A) A representative image and (B) seedling mortality rate (*n*=3) of the wild-type (WT) Dongjin and the *slg-D* mutant at 5 d post-infestation. (C) Mean survival rate (*n*=10) of BPH nymphs feeding on Dongjin (WT) or *slg-D* plants at 2, 4, 6, and 8 d after the start of the infestation. (D) A representative image and (E) seedling mortality rate (*n*=3) of the wild-type (WT) Nipponbare and the *m107* mutant at 5 d post-infestation. (F) Mean survival rate (*n*=10) of BPH nymphs feeding on Nipponbare (WT) or *m107* plants at 2, 4, 6, and 8 d after the start of the infestation. (G) A representative image and (H) seedling mortality rate (*n*=4) of Zhonghua 11 plants after pre-treatment with a mock solution, 1 µM, or 10 µM BL (24-epibrassinolide) applied to plant leaf sheaths for 12 h followed by BPH infestation for 5 d. (I) Mean survival rate (*n*=10) of BPH nymphs feeding on Zhonghua 11 plants treated with BL or mock solution at 2, 4, 6, and 8 d after the start of the infestation. All data are means (±SD). Significant differences were determined using a binomial exact test (B, E, H) or repeated-measures ANOVA with *post hoc* contrasts by Tukey’s test (C, F, I): *P<0.05, **P<0.01.

In view of the significant suppression of the BR pathway upon infestation, we aimed to determine the role of the pathway in the response to BPH attack. A BR-overproducing mutant, *slg-D* (Feng *et al.*, 2016), was chosen for evaluation of BPH resistance and the results showed that it displayed a markedly increased seedling mortality rate after infestation with BPH for 5 d, up to 90% compared with 25% for the WT (Fig. 2A, B). Moreover, BPH nymphs feeding on the mutant plants had higher survival rates than those feeding on WT plants (Dongjin variety) (Fig. 2C). Preference tests showed that the number of BPH nymphs on the mutant was significantly higher than that on WT plants (Supplementary Fig. S2A). To test whether higher BPH susceptibility was caused by internal BR levels, another BR-excessive mutant, *m107* (Wan *et al.*, 2009), was also evaluated. Like *slg-D*, *m107* was also more susceptible to BPH than the equivalent WT plants, and the BPH nymph mortality rate was significant lower on the mutant compared with the WT (Fig. 2D–F). In the preference tests, BPH nymphs were found more often on *m107* than on WT plants (Supplementary Fig. S2B). A BR-deficient mutant, *lhdd10* (Liu *et al.*, 2016), and its WT (9311) were also evaluated for BPH response. As shown in Supplementary Fig. S3, the BPH nymph survival rate was significantly lower on the mutant in comparison with the WT and the number of BPH on *lhdd10* was also significantly lower than those on WT plants, with the result that *lhdd10* was more resistant than the WT. Collectively, these results implied that the increased endogenous BRs led to higher BPH susceptibility, whereas increased resistance occurred when the BR pathway was blocked.

Exogenous BR was applied to seedlings to determine whether BRs negatively regulated the resistance. The mortality rates observed in seedlings pre-treated with 24-epibrassinolide (both 1 μM and 10 μM) were significantly higher than the mock-treated control at 5 d after BPH infestation (Fig. 2G, H). In addition, BPH nymphs feeding on BL-treated plants had higher survival rates than those feeding on control plants (Fig. 2I). These results indicated that BRs promote susceptibility of rice to BPH.

### BRs suppress the SA pathway in rice during BPH infestation

**Fig. 3.**
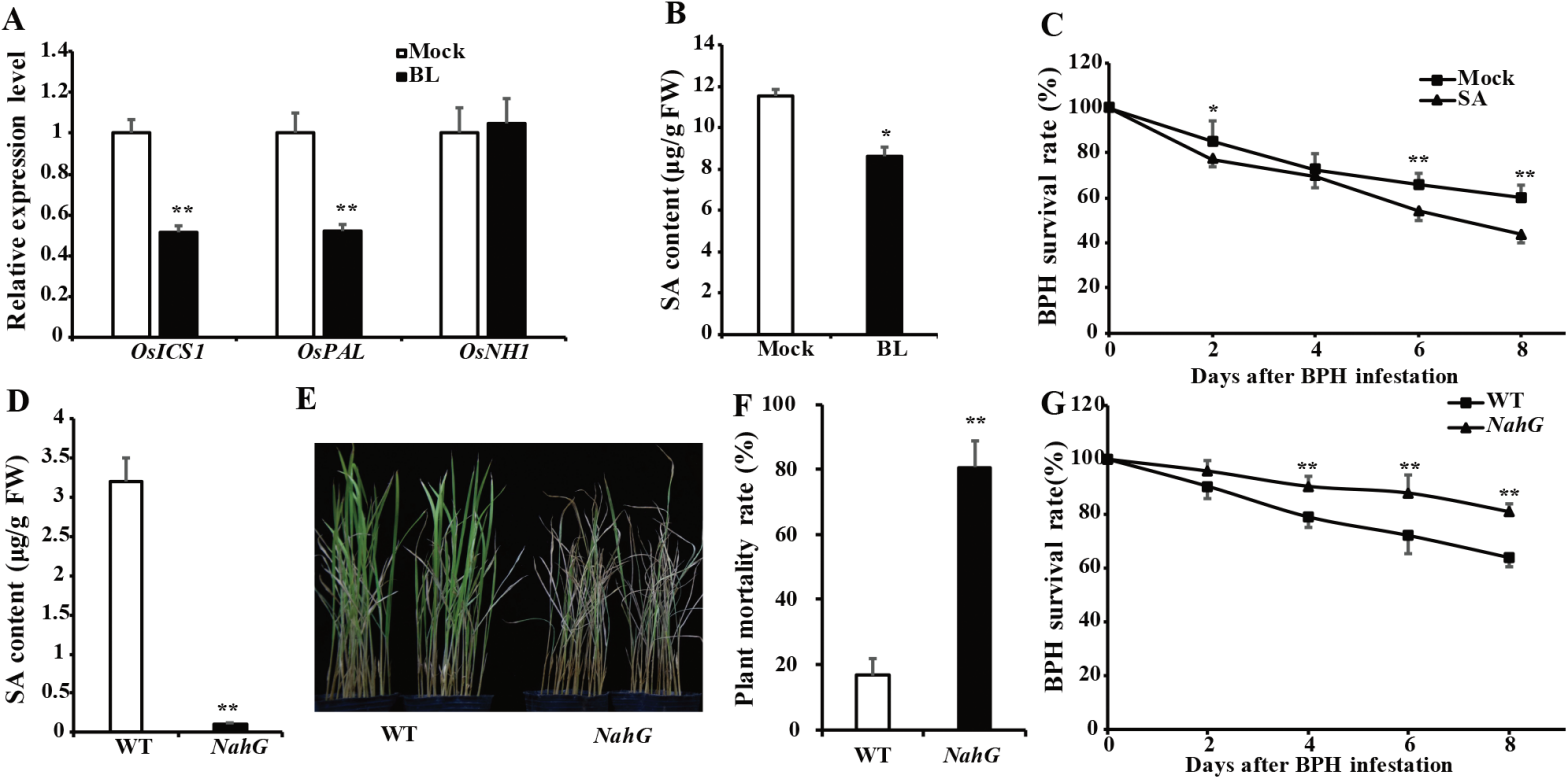
Brassinosteroid (BR)-induced susceptibility to brown planthopper (BPH) involves suppression of salicylic acid (SA)-mediated defense. (A) Transcript analysis of SA-related genes (*OsICS1*, *OsPAL*, and *OsNH1*) and (B) SA content in plants treated with either 24-epibrassinolide (BL) or a mock solution. Zhonghua 11 plants (2 weeks old) were treated by spraying with either 10 µM BL or the mock solution. After 12 h, the plants were infested with BPH for 24 h, and then the leaf sheaths were used for RNA extraction and SA measurement. *Actin* was used as an internal reference. (C) Mean survival rate of BPH nymphs feeding on Zhonghua 11 plants spayed with 0.1 mM SA or a mock solution was recorded at 2, 4, 6, and 8 d after the start of the infestation. (D) SA content of *NahG* and the wild-type (WT) without BPH infestation. (E) A representative image and (F) plant mortality rate of *NahG* and the WT at 5 d after BPH infestation. (G) Mean survival rate (*n*=10) of BPH nymphs feeding on *NahG* and the WT at 2, 4, 6, and 8 d after the start of the infestation. Data are means (±SD), *n*=3 in (A, B, D, F) and *n*=10 in (C, G). Significant differences were determined using one-way ANOVA (A, B, D), repeated-measures ANOVA (C, G) or a binomial exact test (F): *P<0.05, **P<0.01.

Given that the BR-dependent pathway was inhibited while the SA pathway was induced by BPH feeding (Fig. 1, Supplementary Fig. S1A), we further assessed the relationship between BRs and SA in response to BPH infestation. We determined the expression levels of genes related to the SA pathway in leaf sheaths of the BL-treated plants that had been subjected to BPH infestation. The transcript levels of *OsICS1* and *OsPAL* were clearly suppressed in BL-treated plants at 24 h post-infestation, but the levels of *OsNH1* were not significantly different from mock-treated plants (Fig. 3A). A similar pattern was observed in the BR-overproducing mutant (Supplementary Fig. S4A). In agreement with the decrease in SA-related transcripts, SA levels were significantly reduced in seedlings pre-treated with BL and in the BR-overproducing plants at 24 h after BPH infestation, compared with the mock-treated and WT plants (Fig. 3B, Supplementary Fig. S4B). Conversely, transcript levels of *OsICS1* and *OsPAL* as well as SA concentrations were clearly increased in the *lhdd10* mutant at 24 h post-infestation (Supplementary Fig. S4C, D). These results suggested that suppression of SA might be responsible for the enhanced susceptibility to BPH in BL-treated and BR-overproducing plants, and so we investigated the role of SA in the plant response to BPH infestation. As shown in Fig. 3C and Supplementary Fig. S5, the BPH nymphs feeding on SA-treated plants had lower survival rates than those feeding on mock-treated plants, and the mortality rate of SA-treated plants was lower than that of mock plants. On the other hand, SA-deficient plants heterologously expressing the bacterial salicylate hydroxylase gene (*NahG*) were more sensitive to BPH infestation than the WT (Fig. 3D–G), indicating a positive role of SA in response to BPH infestation. Our data thus suggested that BRs negatively regulate BPH resistance through suppressing the SA pathway in rice.

### BRs induce the JA pathway in rice during BPH infestation

**Fig. 4.**
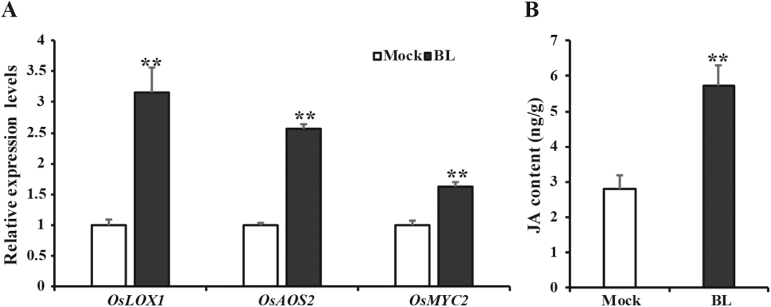
Brassinosteroids (BRs) induced the jasmonic acid (JA) pathway after brown planthopper (BPH) infestation. (A) Transcript analysis of JA-related genes (*OsLOX1*, *OsAOS2*, and *OsMYC2*) in Zhonghua 11 plants treated with either 24-epibrassinolide (BL) or a mock solution. The expression level in rice plants with treated with the mock solution was set as 1.0. (B) Levels of JA in rice stems of mock- and BL-treated plants at 24 h post-infestation. Plants were treated by spraying with either 10 µM BL or a mock solution when they were 2 weeks old. After 12 h, the plants were infested with BPH for 24 h, and then the leaf sheaths were used for RNA extraction and JA measurement. All data are means (±SD), *n*=3. Significant differences were determined using one-way ANOVA: **P<0.01.

In view of the opposite expression patterns of genes related to the BR and JA pathways after BPH infestation (Fig. 1, Supplementary Fig. S1B), we further investigated whether BRs also suppressed the JA pathway. As shown in Fig. 4A, the expression levels of JA-related genes (*OsMYC2*, *OsAOS2*, and *OsLOX1*) were unexpectedly increased at 24 h post-infestation in BL-treated plants. In agreement with the increased expression of JA-related genes, the JA concentration in leaf sheath of BL-treated plants was higher than that in mock-treated plants (5.71 ng g^–1^ versus 2.78 ng g^–1^ FW). The three JA-related genes were also induced in the BR overproducing plants while they were suppressed in BR-deficient plants after BPH infestation (Supplementary Fig. S6). Thus, BRs could induce the JA pathway after BPH infestation.

### BR-mediated suppression of the SA pathway after BPH infestation depends on the JA pathway

**Fig. 5.**
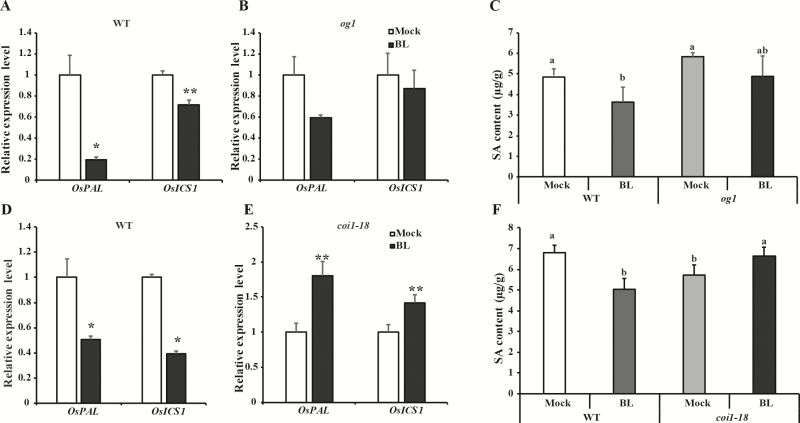
Brassinosteroid (BR)-mediated suppression of salicylic acid (SA) depends on the jasmonic acid (JA) pathway in rice. (A, B, D, E) Following treatment with either 24-epibrassinolide (BL) or a mock solution, genes involved in SA biosynthesis (*OsICS1* and *OsPAL*) were monitored in plants of (A) the 9311 wild-type (WT) and (B) the JA-deficient mutant *og1*; and (D) the Nipponbare WT and (E) the JA-insensitive mutant *coi1-18*. Plants were treated by spraying with either 10 µM BL or the mock solution when they were 2 weeks old. After 12 h, the plants were infested with BPH for 24 h and then expression levels were determined. The expression level in plants with treated with the mock solution was set as 1.0. Data are means (±SD), *n*=3. Significant differences between the BL- and mock-treated plants were determined using one-way ANOVA: *P<0.05, **P<0.01. (C, F) SA content in leaf sheaths of (C) *og1* and WT (9311) plants, and (F) *coi1-18* and WT (Nipponbare) plants after treatment with either BL or a mock solution followed by BPH infestation for 24 h. Data are means (±SD), *n*=3. Different letters indicate significant differences as determined by two-way ANOVA (*P*<0.05).

Previous studies have shown that the JA and SA pathways act in a mutually antagonistic manner (Zhou *et al.*, 2009, 2014; Thaler *et al.*, 2012), and our study indicated that BRs could suppress the SA pathway and activate the JA pathway. We therefore that assumed the BR-mediated suppression of the SA pathway might be associated with the JA pathway. To test this, the JA-deficient mutant *og1* and JA-insensitive mutant *coi1-18* were used to investigate the effect of exogenous BR on the SA pathway. Instead of suppressing the SA pathway in the WT, inhibitory effects of BRs on transcript levels of *OsICS1* and *OsPAL* were abolished in the *og1* mutant at 24 h after BPH infestation (Fig. 5A, B). Consistent with this result, a decrease of SA levels was observed in WT plants after exogenous BR application followed by BPH infestation for 24 h, but this decrease was eliminated in the *og1* mutant (Fig. 5C). Like *og1*, BR-mediated suppression of the SA pathway also was abolished in *coi1-18*, and interestingly the expression levels of *OsPAL* and *OsICS1* as well as SA concentration were increased in *coi1-18* (Fig. 5D–F). These results collectively suggested that suppression of the SA pathway by BRs might be implemented through the JA pathway.

## Discussion

BRs play an important role in the regulation of plant growth and development, as well as in the response to pathogen infection, but their function in defense against insects has rarely been investigated. Here, we addressed the question of whether BRs are involved in defense responses to the phloem-feeding insect the brown planthopper (BPH). In our studies, transcript levels of BR-related genes as well as BR concentrations were significantly decreased after BPH infestation (Fig. 1). In addition, the BR-overproducing mutants *slg-D* and *m107* showed higher susceptibility to BPH, whereas BR-deficient *lhdd10* mutant plants displayed pronounced resistance relative to the WT (Fig. 2A–F, Supplementary Figs S2, S3). Exogenous BR application also increased susceptibility to BPH (Fig. 2G–I). These observations indicated that BRs are negative regulators of innate immunity in plants, which follows the pattern seen in previous studies that showed that BRs mediate susceptibility to *Pythium graminicola*, *rice black streaked dwarf virus*, and *Spodoptera frugiperda* (Campos *et al.*, 2009; De Vleesschauwer *et al.*, 2012; He *et al.*, 2017). BRs have also been found to be positive regulators of defense against the chewing herbivore *Manduca sexta* and the cell-content feeder *Thrips tabaci*, which employ very different feeding methods to the phloem-feeding BPH (Yang *et al.*, 2013; Miyaji *et al.*, 2014). For example, silencing *BRI1* suppresses herbivory-elicited accumulation of jasmonic acid-isoleucine and diterpene glycosides in *Nicotiana attenuata*, resulting in impaired resistance to the insect herbivore *M. sexta* (Yang *et al.*, 2013). In Arabidopsis, *BIL1/BZR-OX* plants have stronger resistance to insect feeding as a result of inducing the JA pathway (Miyaji *et al.*, 2014). These studies indicate that BRs can positively regulate JA-mediated resistance while negatively regulating SA-mediated resistance.

SA is well known for its involvement in innate immune defense against sap-sucking insects and has been reported to associate with *Bph14*- and *Bph29*-mediated insect resistance (Chaman *et al.*, 2003, Du *et al.*, 2009; Wang *et al.*, 2015*b*). In our study, we found that SA-related genes were up-regulated by BPH attack (Supplementary Fig. S1A). SA-deficient plants were more sensitive to BPH infestation than the WT, while exogenous application of SA could enhance resistance to BPH (Fig. 3C–G, Supplementary Fig. S5). Consistent with previous studies (Wang *et al.*, 2015*b*; Li *et al.*, 2017), our results confirmed that SA plays a positive role in the defense response to BPH. In addition, BR overproduction and exogenous BL treatment down-regulated SA-related genes and reduced the SA content while BR biosynthesis-deficient plants increased both the SA concentration and the expression of SA-related genes compared with WT plants after BPH infestation (Fig. 3A, B, Supplementary Fig. S4), indicating that BRs promoted susceptibility by inhibiting SA-mediated immunity to BPH. Antagonistic relationships between BRs and SA during pathogen infection have been investigated in a previous study, which showed that BRs antagonized salicylate-mediated root immunity by suppressing the transcript levels of *OsNH1* (De Vleesschauwer *et al.*, 2012). However, our results indicated that BRs did not actually affect *OsNH1* transcript levels under infestation conditions (Fig. 3A, Supplementary Fig. S4). These discrepancies may be due to the different plant tissues (leaf sheath versus roots) and biotic stresses (insects versus pathogens) used in these studies, and BR-mediated suppression of the SA pathway was independent of *OsNH1* after BPH feeding, which needs to be further investigated.

Compared with the relatively good understanding of interactions between BRs and JA in dicots in response to pathogens and insect attack, information regarding their responses to insect attack in monocots is limited. In contrast to previous findings that have indicated negative effects of BRs on the SA pathway, our data imply that BRs play a positive role in regulating the JA pathway in response to BPH attack. Exogenous BR application and endogenous overproduction of BRs induced the JA pathway, while BR biosynthesis-deficient plants showed suppression of the JA pathway after BPH infestation (Fig. 4, Supplementary Fig. S6). This was consistent with a similar pattern of positive regulation by BRs on the JA pathway that has been described in dicots (Yang *et al.*, 2011, 2013). In Arabidopsis, JA signaling pathways are constitutively activated in the BR-signaling dominant mutant *bil1-1D*/*bzr1-1D* (Miyaji *et al.*, 2014). Likewise, silencing of *BAK1* and *BRI1* results in decreased JA-Ile levels in *N. attenuata* (Yang *et al.*, 2011, 2013).

The antagonistic crosstalk between the SA and JA pathways has been reported to modulate the defense response in rice (Yuan *et al*., 2007; Zhou *et al.*, 2009, 2014, De Vleesschauwer *et al.*, 2013). Exogenous application of JA can dramatically decrease the SA content in rice, which suggests that JA can suppress the SA pathway (Tamaoki *et al.*, 2013). Our results showed that BR treatment increased the JA concentration while decreasing the SA content, and the suppressive effect of BRs on the SA pathway was eliminated in JA-deficient and JA-insensitive mutants (Fig. 5), indicating that BR-dependent induction of JA might contribute to the suppression of SA mediated by BRs. In comparison with the alleviation of BR-mediated suppression of SA in *og-1*, we found that the SA pathway was actually induced in the *coi1-18* mutant after BL treatment (Fig. 5). A possible reason could be that, except for the canonical JA pathway, there may exist an *OsCOI1*-dependent and biosynthesis-independent pathway of suppression of SA mediated by BRs; this hypothesis needs to be further tested. Although the antagonism between SA and JA has been well described in plant biotic stress responses (Thaler *et al.*, 2012), here we have determined for the first time that the BR-mediated suppression of the SA pathway can occur through the JA pathway.

Growth–defense trade-offs are thought to occur in plants as a result of resource restrictions, and hormone crosstalk has emerged as a major factor in the regulation of such trade-offs (Huot *et al.*, 2014). BRs are crucial regulators of plant growth and in our study the BR pathway was clearly inhibited by BPH feeding whereas the defense-related SA and JA pathways were induced (Fig. 1, Supplementary Fig. S1), indicating that the rice host plants might activate a defense response to BPH infestation at the expense of reduced growth. This conclusion is consistent with the findings of a previous study that auxin-, gibberellin-, and BR-related genes were down-regulated while the SA and JA pathways were activated in response to BPH infestation (Wang *et al.*, 2015*a*). Although the SA and JA pathways were induced upon BPH infestation, we also found that the inductions of the two hormones were not simultaneous. The transcript levels of genes related to the SA pathway were moderately up-regulated at 6 h, their expression peaked at about 12 h, and this was followed by a decrease at 24 h, whereas genes related to the JA pathway were most significantly induced at about 24 h after BPH infestation (Supplementary Fig. S1). Similar transcript patterns of the SA and JA pathways in response to BPH infestation have also been described in previous studies (Du *et al.*, 2009; Zhao *et al.*, 2016).

In summary, we found that BRs positively regulated the JA pathway while negatively regulating the SA pathway, which resulted in increased susceptibility of rice plants to BPH. As a defense mechanism against BPH attack, the BR pathway was inhibited whilst the SA pathway was induced, which was followed by activation of the BPH resistance response. Although the underlying mechanisms of the molecular modulation of the interactions between these hormones needs to be further studied, our findings advance our knowledge of the mechanisms involved in the resistance of plants to insect pests.

## Supplementary data

Supplementary data are available at *JXB* online.

Table S1. Primers used for qRT-PCR analysis in this study.

Fig. S1. Expression patterns of genes related to the salicylic acid and jasmonic acid pathways in rice in response to brown planthopper infestation.

Fig. S2. Dynamic changes in brown planthopper populations on two rice BR-overproducing mutants and the wild-types in a feeding preference experiment.

Fig. S3. Comparison of brown planthopper resistance in the *lhdd10* mutant and its wild-type.

Fig. S4. Expression levels of genes related to salicylic acid (SA) and quantification of SA content in rice BR mutants and wild-types after brown planthopper infestation.

Fig. S5. Effect of exogenous salicylic acid treatment on rice defense against brown planthopper.

Fig. S6. Expression levels of genes related to jasmonic acid in rice BR mutants and wild-types after brown planthopper infestation.

Supplementary Material
