# Supplementary material for: Brassinosteroids mediate susceptibility to brown planthopper by integrating with the salicylic acid and jasmonic acid pathways in rice
Source: J Exp Bot. 2018 Jun 8;69(18):4433–42. doi: 10.1093/jxb/ery223 (PMC6093477; doi:10.1093/jxb/ery223)
Supplement: Supplementary Material [file ery223_suppl_supplementary_materials.docx]

| **Primer name** | **Forward (5'-3')** | **Reverse (5'-3')** |
| --- | --- | --- |
| *SLG* | CAAGTTCGACGGGATGGTCTACCT | CTCGCATTTGGAGGAAGTCTTGG |
| *Actin* | TCCATCTTGGCATCTCTCAG | GTACCCGCATCAGGCATCTG |
| *D2* | CCTTTTGGTGGTGGGCAGAG | TGGGGAAGTTGACGATGTGGT |
| *D11* | CAAGGGACAAGCAAGAAGTTTAC | CGATTTCTATGGGCAGACCTC |
| *OsBRI1* | GCAAGGGTATCTGATTTCGGT | CAAGAGTGGACACGCTAAGGT |
| *OsBZR1* | AGATGGTTCCTTTCGTGGAC | AGAATGAAATCGCCCAAATC |
| *OsPAL* | GGACCGACGAGAAGATAGCAA | CATCAGCTAAGCACATCTGTGAACT |
| *OsNH1* | TTTCCGATGGAGGCAAGAG | GCTGTCATCCGAGCTAAGTGTT |
| *OsICS1* | TATGGTGCTATCCGCTTCGAT | CGAGAACCGAGCTCTCTTCAA |
| *OsPR5* | GAGCCAGGACTTCTACGACC | CATGAGATGATGCATTATGGG |
| *OsAOS2* | GTTGACAACAAGCAGTGCCC | CGGAGGTTGAAGCTTTGGTG |
| *OsLOX1* | GTACGCTGGGTTCACAGCTC | TCAGATGGATGTGCTGTTGG |
| *OsJAmyb* | GAGGACCAGAGTGCAAAAGC | CATGGCATCCTTGAACCTCT |
| *OsMYC2* | AGCTCAACCAGCGCTTCTAC | CCTTCTTGAGCGACTCCATC |

Table S1. Primers used for qRT-PCR analysis in this study.

**
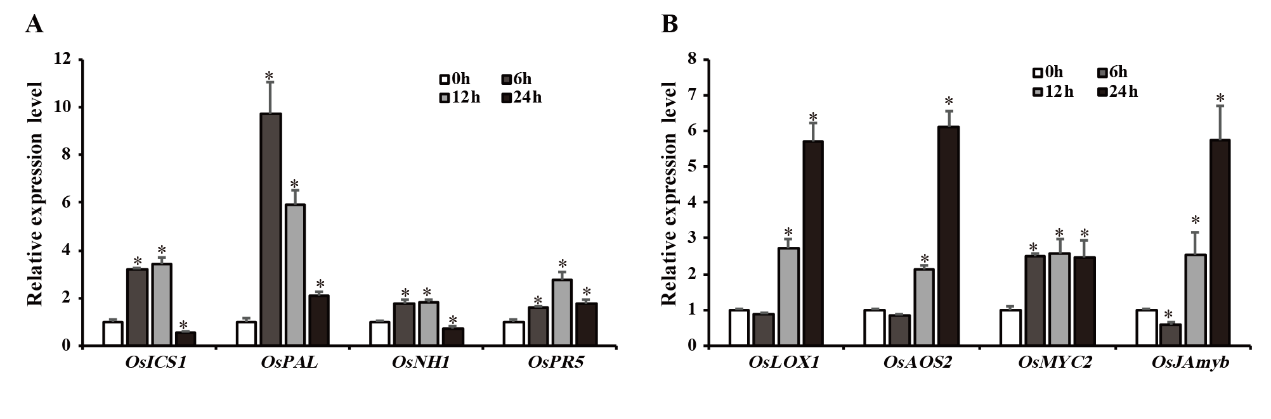
**

Fig. S1 Expression patterns of genes related to the salicylic acid and jasmonic acid pathways in rice in response to brown planthopper infestation. (A) qRT-PCR analysis of SA pathway: SA biosynthesis (*OsPAL* and *OsICS1*) and signaling (*OsNH1* and *OsPR5*) genes. (B) qRT-PCR analysis of JA pathway: JA biosynthesis (*OsLOX1* and *OsAOS2*) and signaling (*OsMYC2* and *OsJAmyb*) genes. Leaf sheaths of 2-week-old Zhonghua 11 plants at 0 h, 6 h, 12 h and 24 h infested with BPH were used for qRT-PCR analyses. *Actin* was used as the internal reference gene. Error bars indicate SD (*n*=3). Each repeat containing a pool of three plants. Expression level at 0 h was set as 1.0. Asterisks on the graphs indicate statistically significant different at *P* < 0.05 in comparison with expression level at 0 h (*n*=3, one-way ANOVA test).


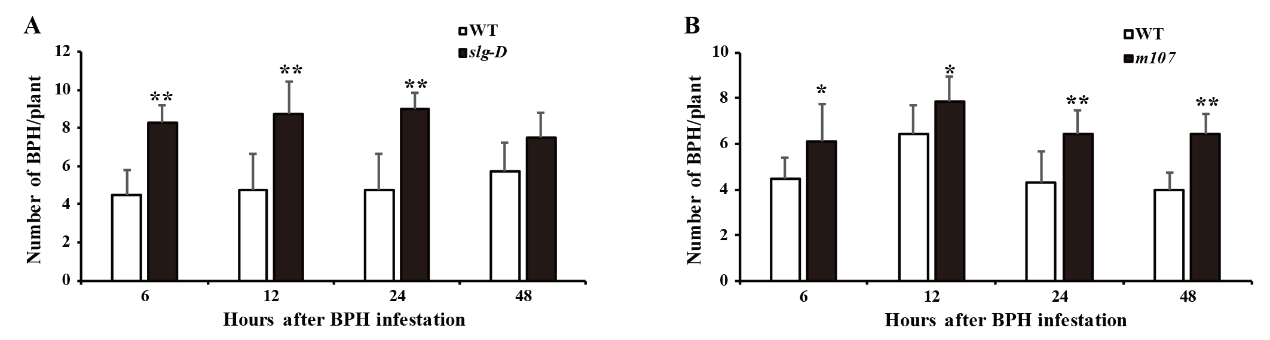


Fig. S2. Dynamic changes in brown planthopper populations on two rice BR-overproducing mutants and the wild-types in a feeding preference experiment. The number of BPH on each variety was recorded at 6, 12, 24 and 48 h post-infestation, respectively. Error bars indicate SD (*n*=8 in A, B). Asterisks indicate statistical signiﬁcance between the WT and the mutant as determined by one-way ANOVA (**P* < 0.05 and ***P* < 0.01).


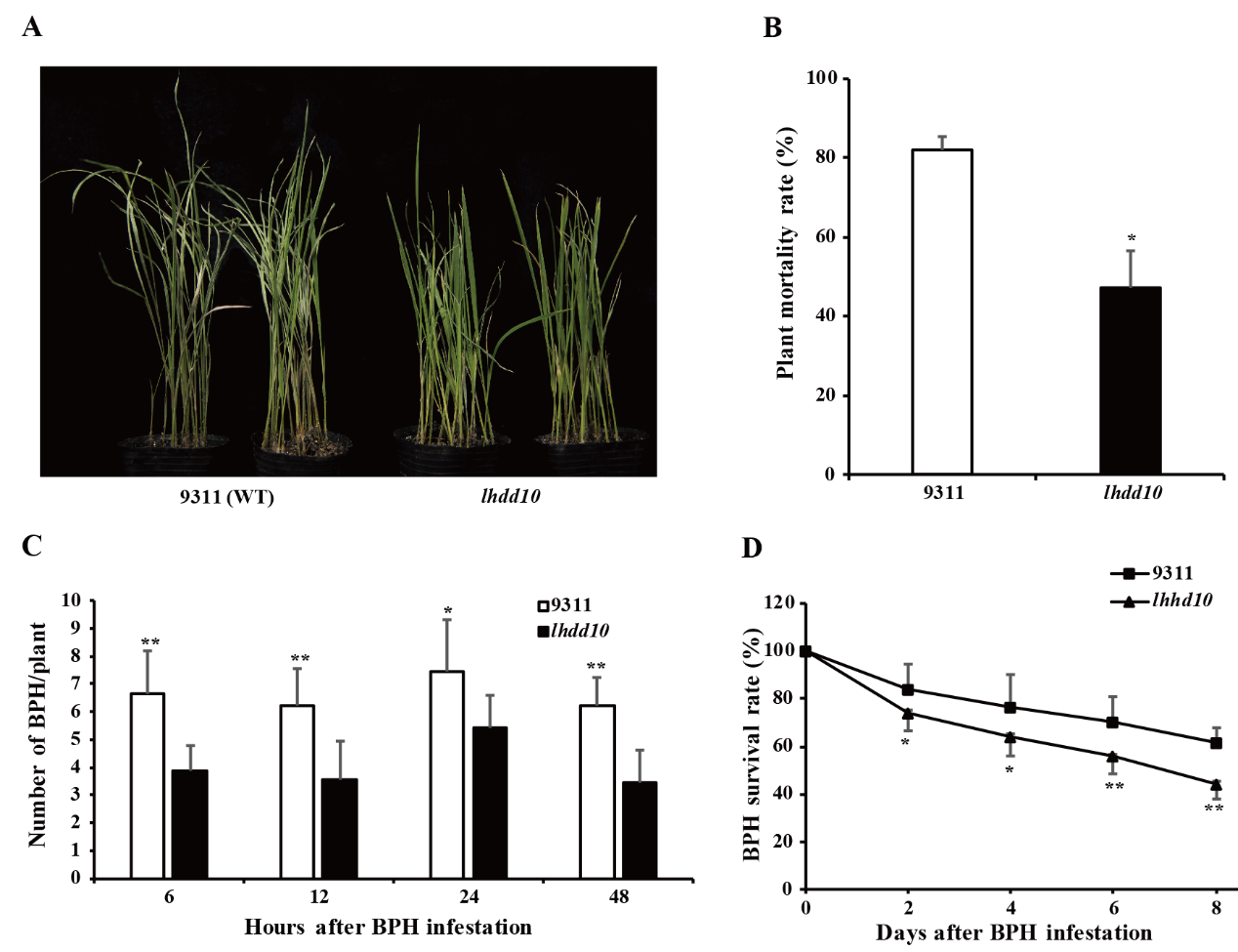


Fig. S3. Comparison of brown planthopper (BPH) resistance in the *lhdd10* mutant and its wild-type. (A) Representative image of 9311 and *lhdd10* at 7 days post-infestation. (B) Seedling mortality rate of 9311 and *lhdd10* at 7 days post-infestation. Error bars indicate SD, *n*=3 independent experiments, **P* < 0.05 (Binomial exact test). (C) Dynamic changes of the brown planthopper (BPH) populations on *lhdd10* and 9311 plants in a feeding preference experiment. The number of BPHs on each variety was recorded at 6, 12, 24 and 48 h post-infestation, respectively. Error bars indicate SD, *n*=3 independent experiments, **P* < 0.05 (one-way ANOVA). (D) Mean survival rate of BPH nymphs that fed on *lhdd10* or 9311 plants at 2, 4, 6 and 8 days after the start of feeding. Error bars indicate SD, *n*=10 independent experiments. Asterisks indicate statistical signiﬁcance between the WT (9311) and *lhdd10* as determined by repeated measures ANOVA (**P* < 0.05; ***P* < 0.01).


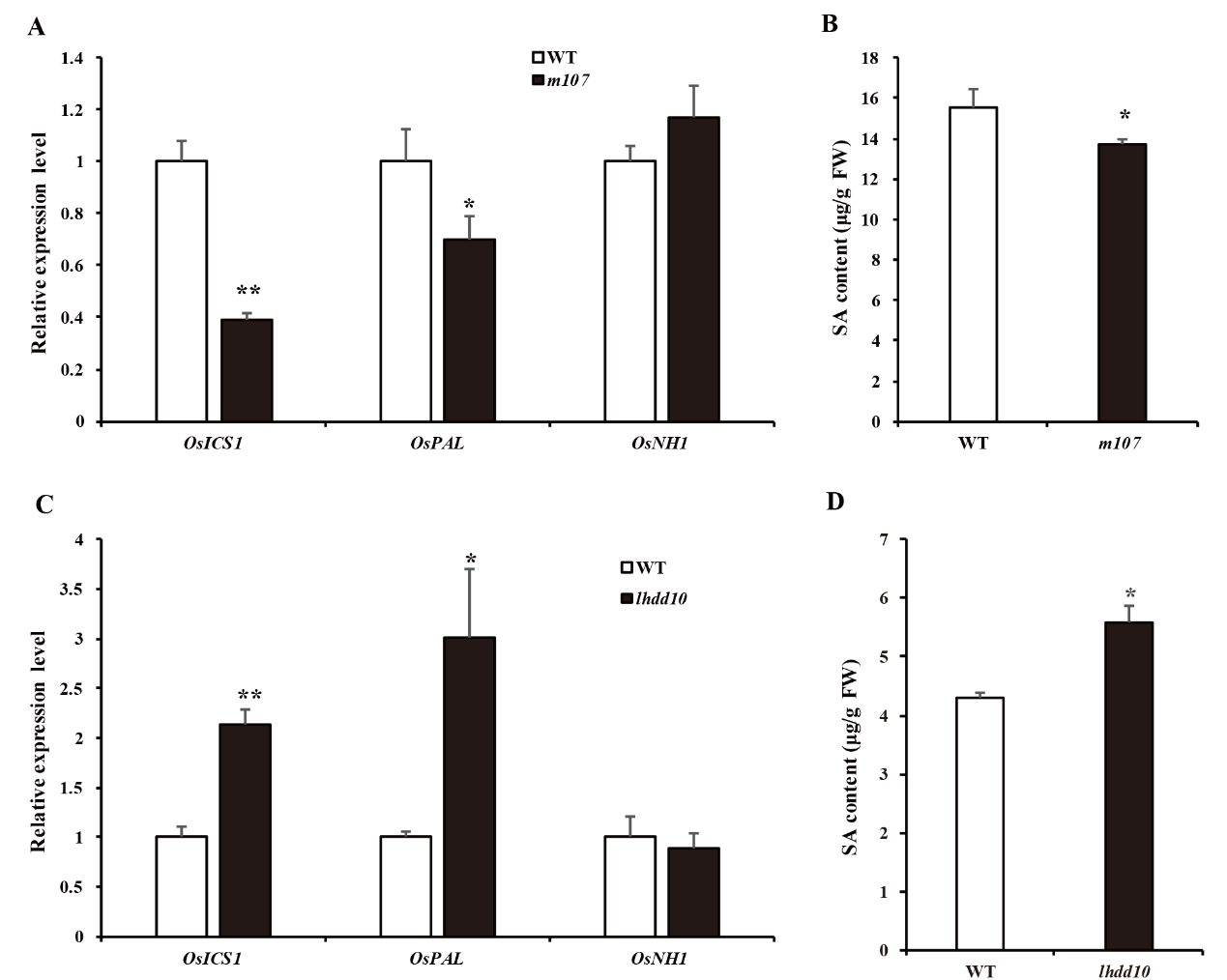


Fig. S4. Expression levels of genes related to salicylic acid (SA) and quantification of SA content in rice BR mutants and wild-types after brown planthopper infestation. Transcript levels of SA-related genes (*OsICS1*, *OsPAL* and *OsNH1*) (A) and SA contents measurement (B) in *m107* and its wild type (WT) plants. Transcript analyses of SA-related genes (*OsICS1*, *OsPAL* and *OsNH1*) (C) and SA contents measurement (D) in *lhhd10* and its wild type (WT) plants. The leaf sheaths of mutants (*m107* and *lhhd10*) and its WT were collected at 24 h after BPH infestation. *m107*, rice BR-overproduction mutant, *lhdd10*, rice BR-deficient mutant. FW, fresh weight. Error bars indicate SD. (*n*=3 in A-D, each containing a pool of three plants, *, *P* < 0.05, by one-way ANOVA).

**
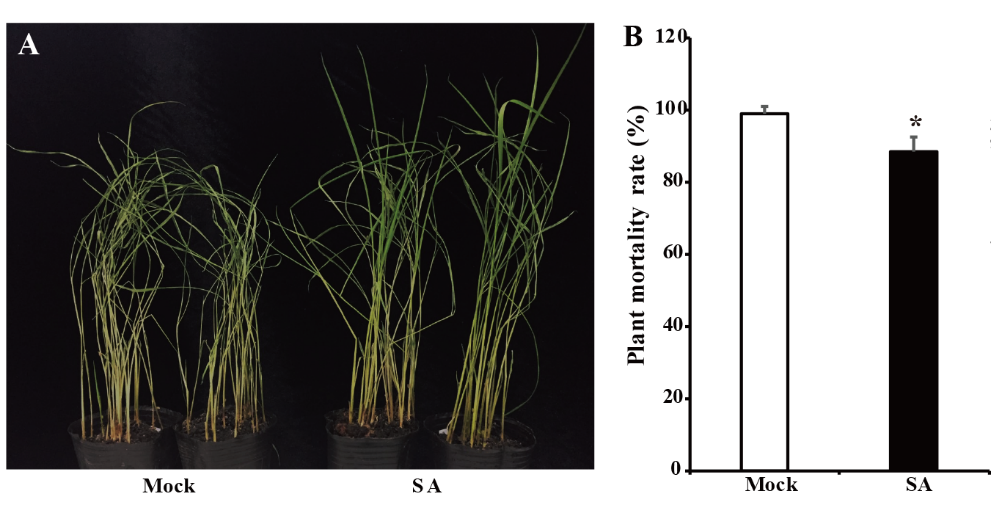
**

Fig. S5. Effect of exogenous salicylic acid treatment on rice defense against brown planthopper. (A) Representative image of SA-treated plants and the mock at 7 days post-infestation. (B) Seedling mortality rate of Zhonghua 11 plants pretreated with the mock and 100 µM SA for 12 h followed by BPH infestation for 7 days. Error bars indicate SD, *n*=3 independent experiments, **P* < 0.05 (Binomial exact test).


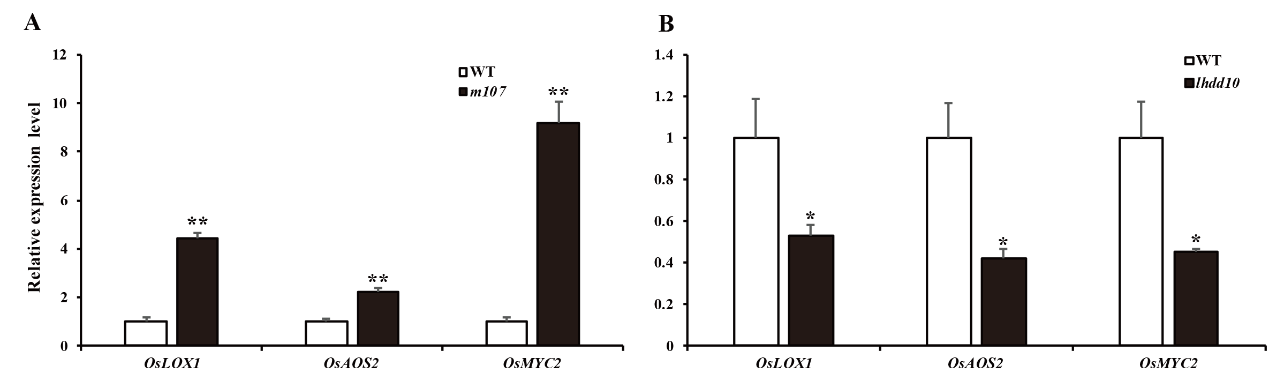


Fig. S6. Expression levels of genes related to jasmonic acid in rice BR mutants and wild-types after brown planthopper infestation. (A) Transcript levels of *OsLOX1*, *OsAOS2* and *OsMYC2* in leaf sheaths of wild type (WT) and *m107* were monitored after BPH infestation for 24 h. (B) Transcript levels of *OsLOX1*, *OsAOS2* and *OsMYC2* in leaf sheaths of WT and *lhhd10* were monitored after BPH infestation for 24 h. *m107*, rice BR-overproduction mutant, *lhdd10*, rice BR-deficient mutant. FW, fresh weight. Expression level in WT plants was set as 1.0. Error bars indicate SD (*n*=3). Each repeat contains a pool of three plants. *Actin* was used as an internal reference. ***, *P* < 0.05 in comparison with the WT plants, respectively (one-way ANOVA).
